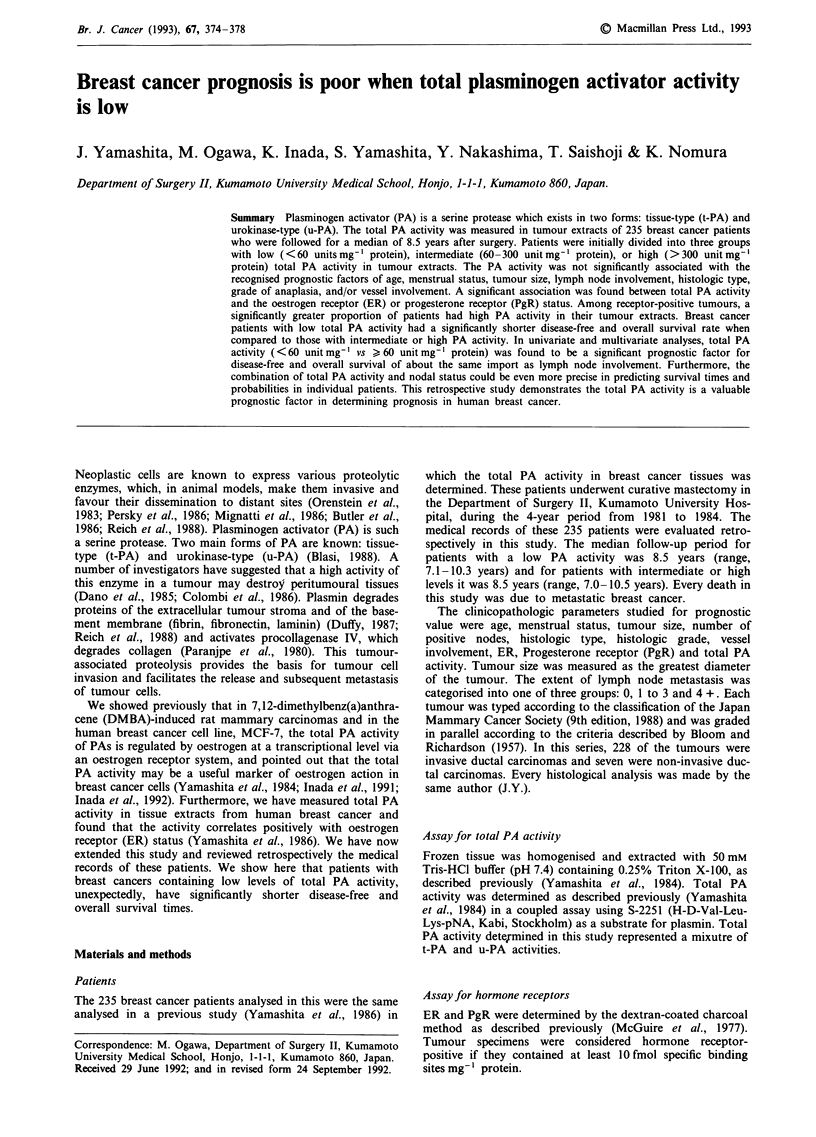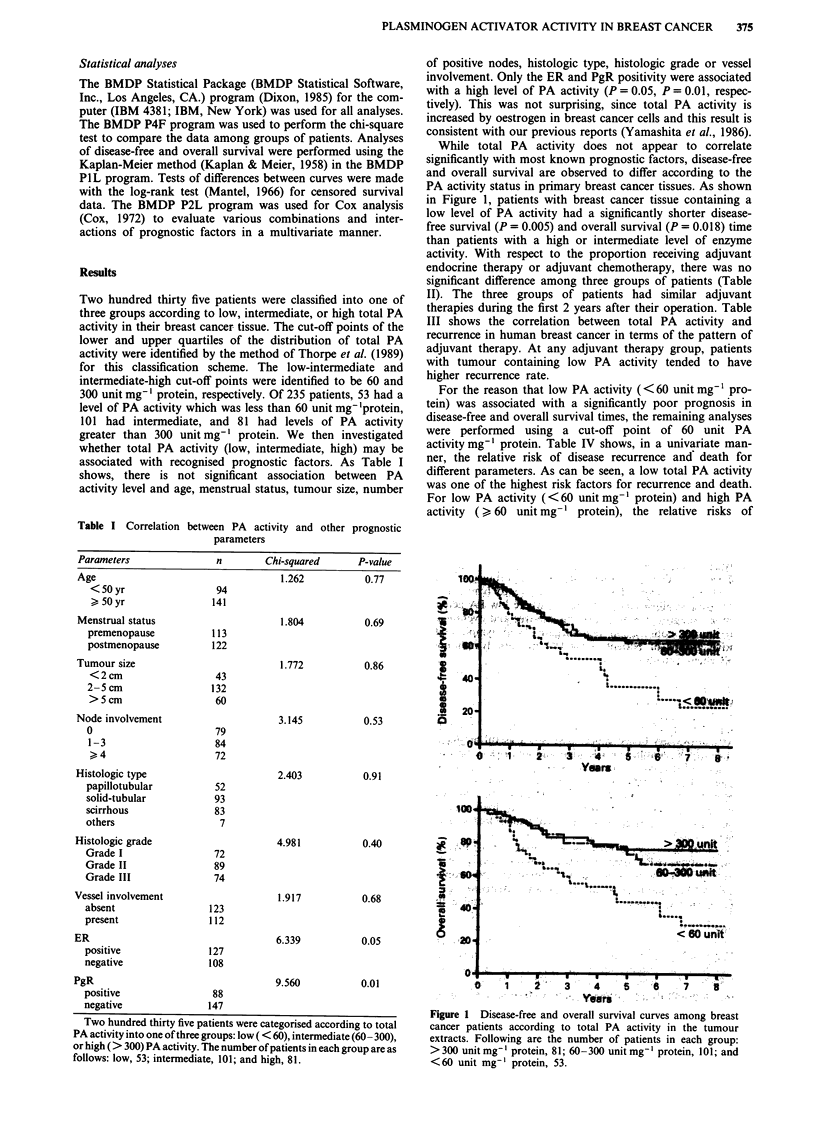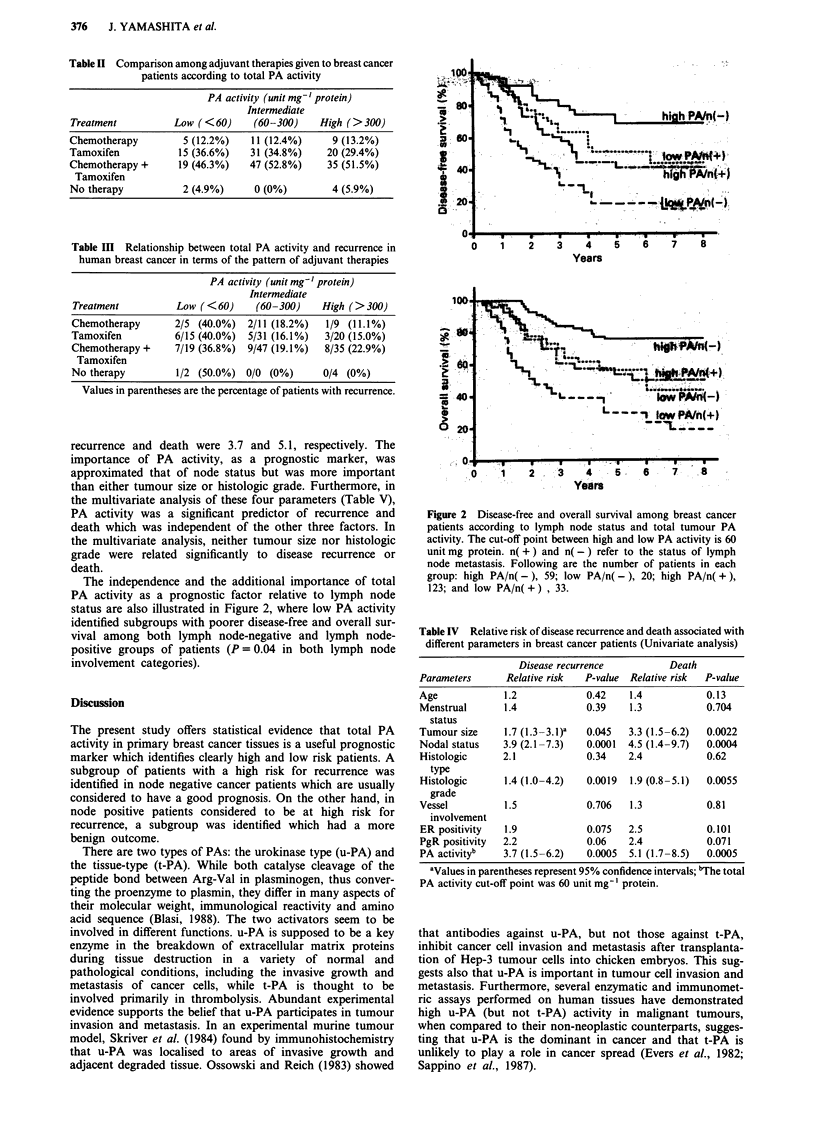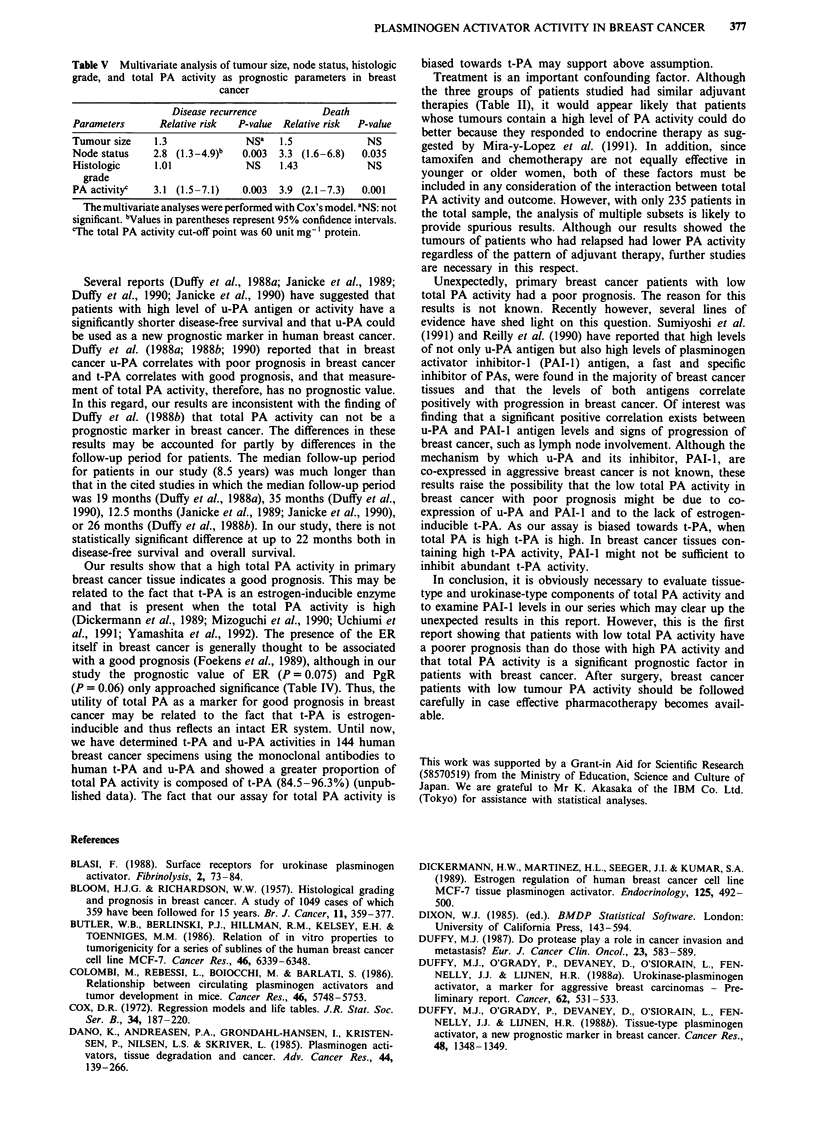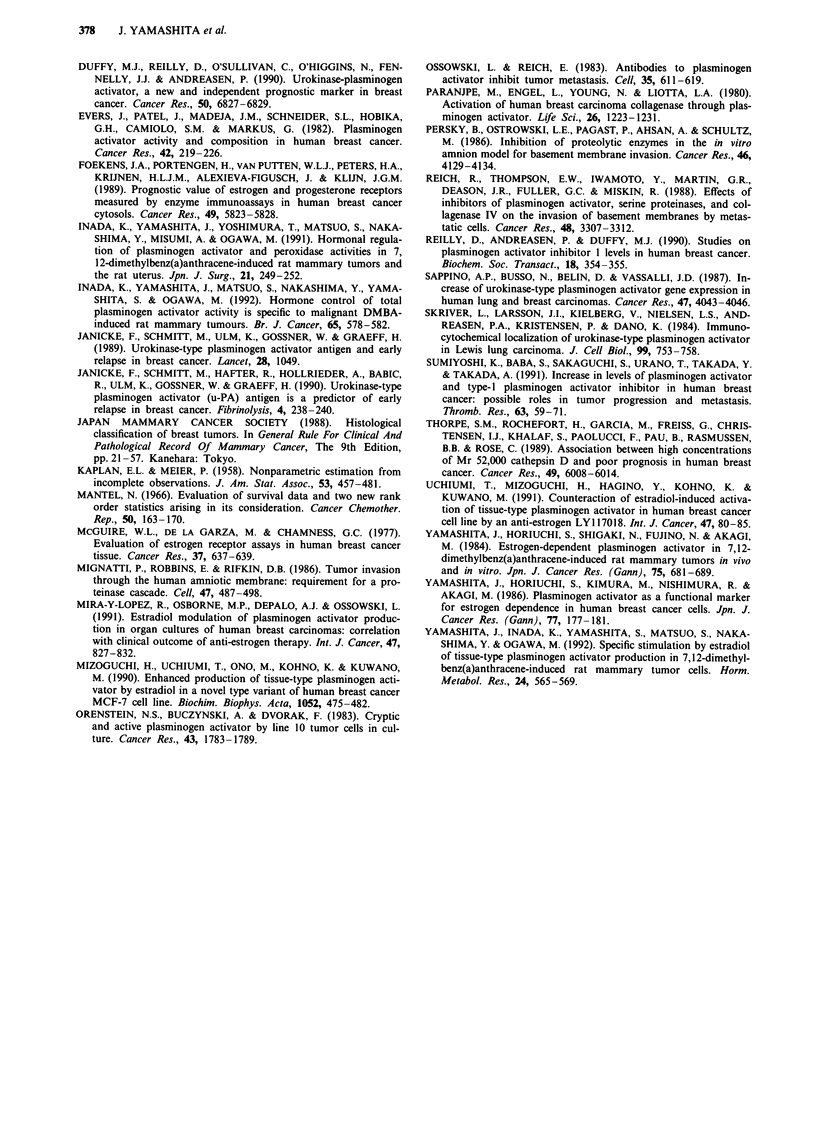# Breast cancer prognosis is poor when total plasminogen activator activity is low.

**DOI:** 10.1038/bjc.1993.68

**Published:** 1993-02

**Authors:** J. Yamashita, M. Ogawa, K. Inada, S. Yamashita, Y. Nakashima, T. Saishoji, K. Nomura

**Affiliations:** Department of Surgery II, Kumamoto University Medical School, Japan.

## Abstract

Plasminogen activator (PA) is a serine protease which exists in two forms: tissue-type (t-PA) and urokinase-type (u-PA). The total PA activity was measured in tumour extracts of 235 breast cancer patients who were followed for a median of 8.5 years after surgery. Patients were initially divided into three groups with low (< 60 units mg-1 protein), intermediate (60-300 unit mg-1 protein), or high (> 300 unit mg-1 protein) total PA activity in tumour extracts. The PA activity was not significantly associated with the recognised prognostic factors of age, menstrual status, tumour size, lymph node involvement, histologic type, grade of anaplasia, and/or vessel involvement. A significant association was found between total PA activity and the oestrogen receptor (ER) or progesterone receptor (PgR) status. Among receptor-positive tumours, a significantly greater proportion of patients had high PA activity in their tumour extracts. Breast cancer patients with low total PA activity had a significantly shorter disease-free and overall survival rate when compared to those with intermediate or high PA activity. In univariate and multivariate analyses, total PA activity (< 60 unit mg-1 vs > or = 60 unit mg-1 protein) was found to be a significant prognostic factor for disease-free and overall survival of about the same import as lymph node involvement. Furthermore, the combination of total PA activity and nodal status could be even more precise in predicting survival times and probabilities in individual patients. This retrospective study demonstrates the total PA activity is a valuable prognostic factor in determining prognosis in human breast cancer.


*Br. J. Cancer* (1993), **67**, 374–378 © Macmillan Press Ltd., 1993

### Breast cancer prognosis is poor when total plasminogen activator activity is low

J. Yamashita, M. Ogawa, K. Inada, S. Yamashita, Y. Nakashima, T. Saishoji & K. Nomura

*Department of Surgery II, Kumamoto University Medical School, Honjo, 1-1-1, Kumamoto 860, Japan.*

**Summary** Plasminogen activator (PA) is a serine protease which exists in two forms: tissue-type (t-PA) and urokinase-type (u-PA). The total PA activity was measured in tumour extracts of 235 breast cancer patients who were followed for a median of 8.5 years after surgery. Patients were initially divided into three groups with low (<60 units $mg^{-1}$ protein), intermediate (60–300 unit $mg^{-1}$ protein), or high (>300 unit $mg^{-1}$ protein) total PA activity in tumour extracts. The PA activity was not significantly associated with the recognised prognostic factors of age, menstrual status, tumour size, lymph node involvement, histologic type, grade of anaplasia, and/or vessel involvement. A significant association was found between total PA activity and the oestrogen receptor (ER) or progesterone receptor (PgR) status. Among receptor-positive tumours, a significantly greater proportion of patients had high PA activity in their tumour extracts. Breast cancer patients with low total PA activity had a significantly shorter disease-free and overall survival rate when compared to those with intermediate or high PA activity. In univariate and multivariate analyses, total PA activity (<60 unit $mg^{-1}$ *vs* ⩾60 unit $mg^{-1}$ protein) was found to be a significant prognostic factor for disease-free and overall survival of about the same import as lymph node involvement. Furthermore, the combination of total PA activity and nodal status could be even more precise in predicting survival times and probabilities in individual patients. This retrospective study demonstrates the total PA activity is a valuable prognostic factor in determining prognosis in human breast cancer.

Neoplastic cells are known to express various proteolytic enzymes, which, in animal models, make them invasive and favour their dissemination to distant sites (Orenstein *et al.*, 1983; Persky *et al.*, 1986; Mignatti *et al.*, 1986; Butler *et al.*, 1986; Reich *et al.*, 1988). Plasminogen activator (PA) is such a serine protease. Two main forms of PA are known: tissue-type (t-PA) and urokinase-type (u-PA) (Blasi, 1988). A number of investigators have suggested that a high activity of this enzyme in a tumour may destroy peritumoural tissues (Dano *et al.*, 1985; Colombi *et al.*, 1986). Plasmin degrades proteins of the extracellular tumour stroma and of the basement membrane (fibrin, fibronectin, laminin) (Duffy, 1987; Reich *et al.*, 1988) and activates procollagenase IV, which degrades collagen (Paranjpe *et al.*, 1980). This tumour-associated proteolysis provides the basis for tumour cell invasion and facilitates the release and subsequent metastasis of tumour cells.

We showed previously that in 7,12-dimethylbenz(a)anthracene (DMBA)-induced rat mammary carcinomas and in the human breast cancer cell line, MCF-7, the total PA activity of PAs is regulated by oestrogen at a transcriptional level via an oestrogen receptor system, and pointed out that the total PA activity may be a useful marker of oestrogen action in breast cancer cells (Yamashita *et al.*, 1984; Inada *et al.*, 1991; Inada *et al.*, 1992). Furthermore, we have measured total PA activity in tissue extracts from human breast cancer and found that the activity correlates positively with oestrogen receptor (ER) status (Yamashita *et al.*, 1986). We have now extended this study and reviewed retrospectively the medical records of these patients. We show here that patients with breast cancers containing low levels of total PA activity, unexpectedly, have significantly shorter disease-free and overall survival times.

### Materials and methods

### Patients

The 235 breast cancer patients analysed in this were the same analysed in a previous study (Yamashita *et al.*, 1986) in which the total PA activity in breast cancer tissues was determined. These patients underwent curative mastectomy in the Department of Surgery II, Kumamoto University Hospital, during the 4-year period from 1981 to 1984. The medical records of these 235 patients were evaluated retrospectively in this study. The median follow-up period for patients with a low PA activity was 8.5 years (range, 7.1–10.3 years) and for patients with intermediate or high levels it was 8.5 years (range, 7.0–10.5 years). Every death in this study was due to metastatic breast cancer.

The clinicopathologic parameters studied for prognostic value were age, menstrual status, tumour size, number of positive nodes, histologic type, histologic grade, vessel involvement, ER, Progesterone receptor (PgR) and total PA activity. Tumour size was measured as the greatest diameter of the tumour. The extent of lymph node metastasis was categorised into one of three groups: 0, 1 to 3 and 4+. Each tumour was typed according to the classification of the Japan Mammary Cancer Society (9th edition, 1988) and was graded in parallel according to the criteria described by Bloom and Richardson (1957). In this series, 228 of the tumours were invasive ductal carcinomas and seven were non-invasive ductal carcinomas. Every histological analysis was made by the same author (J.Y.).

### Assay for total PA activity

Frozen tissue was homogenised and extracted with 50 mM Tris-HCl buffer (pH 7.4) containing 0.25% Triton X-100, as described previously (Yamashita *et al.*, 1984). Total PA activity was determined as described previously (Yamashita *et al.*, 1984) in a coupled assay using S-2251 (H-D-Val-Leu-Lys-pNA, Kabi, Stockholm) as a substrate for plasmin. Total PA activity determined in this study represented a mixutre of t-PA and u-PA activities.

### Assay for hormone receptors

ER and PgR were determined by the dextran-coated charcoal method as described previously (McGuire *et al.*, 1977). Tumour specimens were considered hormone receptor-positive if they contained at least 10 fmol specific binding sites $mg^{-1}$ protein.

Correspondence: M. Ogawa, Department of Surgery II, Kumamoto University Medical School, Honjo, 1-1-1, Kumamoto 860, Japan.
Received 29 June 1992; and in revised form 24 September 1992.

*Statistical analyses*

The BMDP Statistical Package (BMDP Statistical Software, Inc., Los Angeles, CA.) program (Dixon, 1985) for the computer (IBM 4381; IBM, New York) was used for all analyses. The BMDP P4F program was used to perform the chi-square test to compare the data among groups of patients. Analyses of disease-free and overall survival were performed using the Kaplan-Meier method (Kaplan & Meier, 1958) in the BMDP P1L program. Tests of differences between curves were made with the log-rank test (Mantel, 1966) for censored survival data. The BMDP P2L program was used for Cox analysis (Cox, 1972) to evaluate various combinations and interactions of prognostic factors in a multivariate manner.

### Results

Two hundred thirty five patients were classified into one of three groups according to low, intermediate, or high total PA activity in their breast cancer tissue. The cut-off points of the lower and upper quartiles of the distribution of total PA activity were identified by the method of Thorpe *et al.* (1989) for this classification scheme. The low-intermediate and intermediate-high cut-off points were identified to be 60 and 300 unit $mg^{-1}$ protein, respectively. Of 235 patients, 53 had a level of PA activity which was less than 60 unit $mg^{-1}$protein, 101 had intermediate, and 81 had levels of PA activity greater than 300 unit $mg^{-1}$ protein. We then investigated whether total PA activity (low, intermediate, high) may be associated with recognised prognostic factors. As Table I shows, there is not significant association between PA activity level and age, menstrual status, tumour size, number of positive nodes, histologic type, histologic grade or vessel involvement. Only the ER and PgR positivity were associated with a high level of PA activity ($P = 0.05$, $P = 0.01$, respectively). This was not surprising, since total PA activity is increased by oestrogen in breast cancer cells and this result is consistent with our previous reports (Yamashita *et al.*, 1986).

While total PA activity does not appear to correlate significantly with most known prognostic factors, disease-free and overall survival are observed to differ according to the PA activity status in primary breast cancer tissues. As shown in Figure 1, patients with breast cancer tissue containing a low level of PA activity had a significantly shorter disease-free survival ($P = 0.005$) and overall survival ($P = 0.018$) time than patients with a high or intermediate level of enzyme activity. With respect to the proportion receiving adjuvant endocrine therapy or adjuvant chemotherapy, there was no significant difference among three groups of patients (Table II). The three groups of patients had similar adjuvant therapies during the first 2 years after their operation. Table III shows the correlation between total PA activity and recurrence in human breast cancer in terms of the pattern of adjuvant therapy. At any adjuvant therapy group, patients with tumour containing low PA activity tended to have higher recurrence rate.

For the reason that low PA activity ($<60$ unit $mg^{-1}$ protein) was associated with a significantly poor prognosis in disease-free and overall survival times, the remaining analyses were performed using a cut-off point of 60 unit PA activity $mg^{-1}$ protein. Table IV shows, in a univariate manner, the relative risk of disease recurrence and death for different parameters. As can be seen, a low total PA activity was one of the highest risk factors for recurrence and death. For low PA activity ($<60$ unit $mg^{-1}$ protein) and high PA activity ($\geqslant 60$ unit $mg^{-1}$ protein), the relative risks of

**Table I** Correlation between PA activity and other prognostic parameters

| *Parameters* | *n* | *Chi-squared* | *P-value* |
|---|---|---|---|
| Age | | 1.262 | 0.77 |
| $<50$ yr | 94 | | |
| $\geqslant 50$ yr | 141 | | |
| Menstrual status | | 1.804 | 0.69 |
| premenopause | 113 | | |
| postmenopause | 122 | | |
| Tumour size | | 1.772 | 0.86 |
| $<2$ cm | 43 | | |
| 2–5 cm | 132 | | |
| $>5$ cm | 60 | | |
| Node involvement | | 3.145 | 0.53 |
| 0 | 79 | | |
| 1–3 | 84 | | |
| $\geqslant 4$ | 72 | | |
| Histologic type | | 2.403 | 0.91 |
| papillotubular | 52 | | |
| solid-tubular | 93 | | |
| scirrhous | 83 | | |
| others | 7 | | |
| Histologic grade | | 4.981 | 0.40 |
| Grade I | 72 | | |
| Grade II | 89 | | |
| Grade III | 74 | | |
| Vessel involvement | | 1.917 | 0.68 |
| absent | 123 | | |
| present | 112 | | |
| ER | | 6.339 | 0.05 |
| positive | 127 | | |
| negative | 108 | | |
| PgR | | 9.560 | 0.01 |
| positive | 88 | | |
| negative | 147 | | |

Two hundred thirty five patients were categorised according to total PA activity into one of three groups: low ($<60$), intermediate (60–300), or high ($>300$) PA activity. The number of patients in each group are as follows: low, 53; intermediate, 101; and high, 81.

**Figure 1** Disease-free and overall survival curves among breast cancer patients according to total PA activity in the tumour extracts. Following are the number of patients in each group: $>300$ unit $mg^{-1}$ protein, 81; 60–300 unit $mg^{-1}$ protein, 101; and $<60$ unit $mg^{-1}$ protein, 53.

**Table II** Comparison among adjuvant therapies given to breast cancer patients according to total PA activity

| Treatment | PA activity (unit $mg^{-1}$ protein) Low (<60) | Intermediate (60–300) | High (>300) |
|---|---|---|---|
| Chemotherapy | 5 (12.2%) | 11 (12.4%) | 9 (13.2%) |
| Tamoxifen | 15 (36.6%) | 31 (34.8%) | 20 (29.4%) |
| Chemotherapy + Tamoxifen | 19 (46.3%) | 47 (52.8%) | 35 (51.5%) |
| No therapy | 2 (4.9%) | 0 (0%) | 4 (5.9%) |

**Table III** Relationship between total PA activity and recurrence in human breast cancer in terms of the pattern of adjuvant therapies

| Treatment | PA activity (unit $mg^{-1}$ protein) Low (<60) | Intermediate (60–300) | High (>300) |
|---|---|---|---|
| Chemotherapy | 2/5 (40.0%) | 2/11 (18.2%) | 1/9 (11.1%) |
| Tamoxifen | 6/15 (40.0%) | 5/31 (16.1%) | 3/20 (15.0%) |
| Chemotherapy + Tamoxifen | 7/19 (36.8%) | 9/47 (19.1%) | 8/35 (22.9%) |
| No therapy | 1/2 (50.0%) | 0/0 (0%) | 0/4 (0%) |

Values in parentheses are the percentage of patients with recurrence.

recurrence and death were 3.7 and 5.1, respectively. The importance of PA activity, as a prognostic marker, was approximated that of node status but was more important than either tumour size or histologic grade. Furthermore, in the multivariate analysis of these four parameters (Table V), PA activity was a significant predictor of recurrence and death which was independent of the other three factors. In the multivariate analysis, neither tumour size nor histologic grade were related significantly to disease recurrence or death.

The independence and the additional importance of total PA activity as a prognostic factor relative to lymph node status are also illustrated in Figure 2, where low PA activity identified subgroups with poorer disease-free and overall survival among both lymph node-negative and lymph node-positive groups of patients ($P = 0.04$ in both lymph node involvement categories).

### Discussion

The present study offers statistical evidence that total PA activity in primary breast cancer tissues is a useful prognostic marker which identifies clearly high and low risk patients. A subgroup of patients with a high risk for recurrence was identified in node negative cancer patients which are usually considered to have a good prognosis. On the other hand, in node positive patients considered to be at high risk for recurrence, a subgroup was identified which had a more benign outcome.

There are two types of PAs: the urokinase type (u-PA) and the tissue-type (t-PA). While both catalyse cleavage of the peptide bond between Arg-Val in plasminogen, thus converting the proenzyme to plasmin, they differ in many aspects of their molecular weight, immunological reactivity and amino acid sequence (Blasi, 1988). The two activators seem to be involved in different functions. u-PA is supposed to be a key enzyme in the breakdown of extracellular matrix proteins during tissue destruction in a variety of normal and pathological conditions, including the invasive growth and metastasis of cancer cells, while t-PA is thought to be involved primarily in thrombolysis. Abundant experimental evidence supports the belief that u-PA participates in tumour invasion and metastasis. In an experimental murine tumour model, Skriver *et al.* (1984) found by immunohistochemistry that u-PA was localised to areas of invasive growth and adjacent degraded tissue. Ossowski and Reich (1983) showed

**Figure 2** Disease-free and overall survival among breast cancer patients according to lymph node status and total tumour PA activity. The cut-off point between high and low PA activity is 60 unit mg protein. n(+) and n(−) refer to the status of lymph node metastasis. Following are the number of patients in each group: high PA/n(−), 59; low PA/n(−), 20; high PA/n(+), 123; and low PA/n(+), 33.

**Table IV** Relative risk of disease recurrence and death associated with different parameters in breast cancer patients (Univariate analysis)

| Parameters | Disease recurrence Relative risk | P-value | Death Relative risk | P-value |
|---|---|---|---|---|
| Age | 1.2 | 0.42 | 1.4 | 0.13 |
| Menstrual status | 1.4 | 0.39 | 1.3 | 0.704 |
| Tumour size | 1.7 (1.3–3.1)[a] | 0.045 | 3.3 (1.5–6.2) | 0.0022 |
| Nodal status | 3.9 (2.1–7.3) | 0.0001 | 4.5 (1.4–9.7) | 0.0004 |
| Histologic type | 2.1 | 0.34 | 2.4 | 0.62 |
| Histologic grade | 1.4 (1.0–4.2) | 0.0019 | 1.9 (0.8–5.1) | 0.0055 |
| Vessel involvement | 1.5 | 0.706 | 1.3 | 0.81 |
| ER positivity | 1.9 | 0.075 | 2.5 | 0.101 |
| PgR positivity | 2.2 | 0.06 | 2.4 | 0.071 |
| PA activity[b] | 3.7 (1.5–6.2) | 0.0005 | 5.1 (1.7–8.5) | 0.0005 |

[a]Values in parentheses represent 95% confidence intervals; [b]The total PA activity cut-off point was 60 unit $mg^{-1}$ protein.

that antibodies against u-PA, but not those against t-PA, inhibit cancer cell invasion and metastasis after transplantation of Hep-3 tumour cells into chicken embryos. This suggests also that u-PA is important in tumour cell invasion and metastasis. Furthermore, several enzymatic and immunometric assays performed on human tissues have demonstrated high u-PA (but not t-PA) activity in malignant tumours, when compared to their non-neoplastic counterparts, suggesting that u-PA is the dominant in cancer and that t-PA is unlikely to play a role in cancer spread (Evers *et al.*, 1982; Sappino *et al.*, 1987).

**Table V** Multivariate analysis of tumour size, node status, histologic grade, and total PA activity as prognostic parameters in breast cancer

| | Disease recurrence | | Death | |
|---|---|---|---|---|
| *Parameters* | *Relative risk* | *P-value* | *Relative risk* | *P-value* |
| Tumour size | 1.3 | NS[a] | 1.5 | NS |
| Node status | 2.8 (1.3–4.9)[b] | 0.003 | 3.3 (1.6–6.8) | 0.035 |
| Histologic grade | 1.01 | NS | 1.43 | NS |
| PA activity[c] | 3.1 (1.5–7.1) | 0.003 | 3.9 (2.1–7.3) | 0.001 |

The multivariate analyses were performed with Cox's model. [a]NS: not significant. [b]Values in parentheses represent 95% confidence intervals. [c]The total PA activity cut-off point was 60 unit $mg^{-1}$ protein.

Several reports (Duffy *et al.*, 1988*a*; Janicke *et al.*, 1989; Duffy *et al.*, 1990; Janicke *et al.*, 1990) have suggested that patients with high level of u-PA antigen or activity have a significantly shorter disease-free survival and that u-PA could be used as a new prognostic marker in human breast cancer. Duffy *et al.* (1988*a*; 1988*b*; 1990) reported that in breast cancer u-PA correlates with poor prognosis in breast cancer and t-PA correlates with good prognosis, and that measurement of total PA activity, therefore, has no prognostic value. In this regard, our results are inconsistent with the finding of Duffy *et al.* (1988*b*) that total PA activity can not be a prognostic marker in breast cancer. The differences in these results may be accounted for partly by differences in the follow-up period for patients. The median follow-up period for patients in our study (8.5 years) was much longer than that in the cited studies in which the median follow-up period was 19 months (Duffy *et al.*, 1988*a*), 35 months (Duffy *et al.*, 1990), 12.5 months (Janicke *et al.*, 1989; Janicke *et al.*, 1990), or 26 months (Duffy *et al.*, 1988*b*). In our study, there is not statistically significant difference at up to 22 months both in disease-free survival and overall survival.

Our results show that a high total PA activity in primary breast cancer tissue indicates a good prognosis. This may be related to the fact that t-PA is an estrogen-inducible enzyme and that is present when the total PA activity is high (Dickermann *et al.*, 1989; Mizoguchi *et al.*, 1990; Uchiumi *et al.*, 1991; Yamashita *et al.*, 1992). The presence of the ER itself in breast cancer is generally thought to be associated with a good prognosis (Foekens *et al.*, 1989), although in our study the prognostic value of ER ($P = 0.075$) and PgR ($P = 0.06$) only approached significance (Table IV). Thus, the utility of total PA as a marker for good prognosis in breast cancer may be related to the fact that t-PA is estrogen-inducible and thus reflects an intact ER system. Until now, we have determined t-PA and u-PA activities in 144 human breast cancer specimens using the monoclonal antibodies to human t-PA and u-PA and showed a greater proportion of total PA activity is composed of t-PA (84.5–96.3%) (unpublished data). The fact that our assay for total PA activity is biased towards t-PA may support above assumption.

Treatment is an important confounding factor. Although the three groups of patients studied had similar adjuvant therapies (Table II), it would appear likely that patients whose tumours contain a high level of PA activity could do better because they responded to endocrine therapy as suggested by Mira-y-Lopez *et al.* (1991). In addition, since tamoxifen and chemotherapy are not equally effective in younger or older women, both of these factors must be included in any consideration of the interaction between total PA activity and outcome. However, with only 235 patients in the total sample, the analysis of multiple subsets is likely to provide spurious results. Although our results showed the tumours of patients who had relapsed had lower PA activity regardless of the pattern of adjuvant therapy, further studies are necessary in this respect.

Unexpectedly, primary breast cancer patients with low total PA activity had a poor prognosis. The reason for this results is not known. Recently however, several lines of evidence have shed light on this question. Sumiyoshi *et al.* (1991) and Reilly *et al.* (1990) have reported that high levels of not only u-PA antigen but also high levels of plasminogen activator inhibitor-1 (PAI-1) antigen, a fast and specific inhibitor of PAs, were found in the majority of breast cancer tissues and that the levels of both antigens correlate positively with progression in breast cancer. Of interest was finding that a significant positive correlation exists between u-PA and PAI-1 antigen levels and signs of progression of breast cancer, such as lymph node involvement. Although the mechanism by which u-PA and its inhibitor, PAI-1, are co-expressed in aggressive breast cancer is not known, these results raise the possibility that the low total PA activity in breast cancer with poor prognosis might be due to co-expression of u-PA and PAI-1 and to the lack of estrogen-inducible t-PA. As our assay is biased towards t-PA, when total PA is high t-PA is high. In breast cancer tissues containing high t-PA activity, PAI-1 might not be sufficient to inhibit abundant t-PA activity.

In conclusion, it is obviously necessary to evaluate tissue-type and urokinase-type components of total PA activity and to examine PAI-1 levels in our series which may clear up the unexpected results in this report. However, this is the first report showing that patients with low total PA activity have a poorer prognosis than do those with high PA activity and that total PA activity is a significant prognostic factor in patients with breast cancer. After surgery, breast cancer patients with low tumour PA activity should be followed carefully in case effective pharmacotherapy becomes available.

This work was supported by a Grant-in Aid for Scientific Research (58570519) from the Ministry of Education, Science and Culture of Japan. We are grateful to Mr K. Akasaka of the IBM Co. Ltd. (Tokyo) for assistance with statistical analyses.

### References

BLASI, F. (1988). Surface receptors for urokinase plasminogen activator. *Fibrinolysis*, **2**, 73–84.

BLOOM, H.J.G. & RICHARDSON, W.W. (1957). Histological grading and prognosis in breast cancer. A study of 1049 cases of which 359 have been followed for 15 years. *Br. J. Cancer*, **11**, 359–377.

BUTLER, W.B., BERLINSKI, P.J., HILLMAN, R.M., KELSEY, E.H. & TOENNIGES, M.M. (1986). Relation of in vitro properties to tumorigenicity for a series of sublines of the human breast cancer cell line MCF-7. *Cancer Res.*, **46**, 6339–6348.

COLOMBI, M., REBESSI, L., BOIOCCHI, M. & BARLATI, S. (1986). Relationship between circulating plasminogen activators and tumor development in mice. *Cancer Res.*, **46**, 5748–5753.

COX, D.R. (1972). Regression models and life tables. *J.R. Stat. Soc. Ser. B.*, **34**, 187–220.

DANO, K., ANDREASEN, P.A., GRONDAHL-HANSEN, I., KRISTENSEN, P., NILSEN, L.S. & SKRIVER, L. (1985). Plasminogen activators, tissue degradation and cancer. *Adv. Cancer Res.*, **44**, 139–266.

DICKERMANN, H.W., MARTINEZ, H.L., SEEGER, J.I. & KUMAR, S.A. (1989). Estrogen regulation of human breast cancer cell line MCF-7 tissue plasminogen activator. *Endocrinology*, **125**, 492–500.

DIXON, W.J. (1985). (ed.). *BMDP Statistical Software*. London: University of California Press, 143–594.

DUFFY, M.J. (1987). Do protease play a role in cancer invasion and metastasis? *Eur. J. Cancer Clin. Oncol.*, **23**, 583–589.

DUFFY, M.J., O'GRADY, P., DEVANEY, D., O'SIORAIN, L., FENNELLY, J.J. & LIJNEN, H.R. (1988*a*). Urokinase-plasminogen activator, a marker for aggressive breast carcinomas – Preliminary report. *Cancer*, **62**, 531–533.

DUFFY, M.J., O'GRADY, P., DEVANEY, D., O'SIORAIN, L., FENNELLY, J.J. & LIJNEN, H.R. (1988*b*). Tissue-type plasminogen activator, a new prognostic marker in breast cancer. *Cancer Res.*, **48**, 1348–1349.

DUFFY, M.J., REILLY, D., O'SULLIVAN, C., O'HIGGINS, N., FENNELLY, J.J. & ANDREASEN, P. (1990). Urokinase-plasminogen activator, a new and independent prognostic marker in breast cancer. *Cancer Res.*, **50**, 6827-6829.

EVERS, J., PATEL, J., MADEJA, J.M., SCHNEIDER, S.L., HOBIKA, G.H., CAMIOLO, S.M. & MARKUS, G. (1982). Plasminogen activator activity and composition in human breast cancer. *Cancer Res.*, **42**, 219-226.

FOEKENS, J.A., PORTENGEN, H., VAN PUTTEN, W.L.J., PETERS, H.A., KRIJNEN, H.L.J.M., ALEXIEVA-FIGUSCH, J. & KLIJN, J.G.M. (1989). Prognostic value of estrogen and progesterone receptors measured by enzyme immunoassays in human breast cancer cytosols. *Cancer Res.*, **49**, 5823-5828.

INADA, K., YAMASHITA, J., YOSHIMURA, T., MATSUO, S., NAKASHIMA, Y., MISUMI, A. & OGAWA, M. (1991). Hormonal regulation of plasminogen activator and peroxidase activities in 7, 12-dimethylbenz(a)anthracene-induced rat mammary tumors and the rat uterus. *Jpn. J. Surg.*, **21**, 249-252.

INADA, K., YAMASHITA, J., MATSUO, S., NAKASHIMA, Y., YAMASHITA, S. & OGAWA, M. (1992). Hormone control of total plasminogen activator activity is specific to malignant DMBA-induced rat mammary tumours. *Br. J. Cancer*, **65**, 578-582.

JANICKE, F., SCHMITT, M., ULM, K., GOSSNER, W. & GRAEFF, H. (1989). Urokinase-type plasminogen activator antigen and early relapse in breast cancer. *Lancet*, **28**, 1049.

JANICKE, F., SCHMITT, M., HAFTER, R., HOLLRIEDER, A., BABIC, R., ULM, K., GOSSNER, W. & GRAEFF, H. (1990). Urokinase-type plasminogen activator (u-PA) antigen is a predictor of early relapse in breast cancer. *Fibrinolysis*, **4**, 238-240.

JAPAN MAMMARY CANCER SOCIETY (1988). Histological classification of breast tumors. In *General Rule For Clinical And Pathological Record Of Mammary Cancer*, The 9th Edition, pp. 21-57. Kanehara: Tokyo.

KAPLAN, E.L. & MEIER, P. (1958). Nonparametric estimation from incomplete observations. *J. Am. Stat. Assoc.*, **53**, 457-481.

MANTEL, N. (1966). Evaluation of survival data and two new rank order statistics arising in its consideration. *Cancer Chemother. Rep.*, **50**, 163-170.

MCGUIRE, W.L., DE LA GARZA, M. & CHAMNESS, G.C. (1977). Evaluation of estrogen receptor assays in human breast cancer tissue. *Cancer Res.*, **37**, 637-639.

MIGNATTI, P., ROBBINS, E. & RIFKIN, D.B. (1986). Tumor invasion through the human amniotic membrane: requirement for a proteinase cascade. *Cell*, **47**, 487-498.

MIRA-Y-LOPEZ, R., OSBORNE, M.P., DEPALO, A.J. & OSSOWSKI, L. (1991). Estradiol modulation of plasminogen activator production in organ cultures of human breast carcinomas: correlation with clinical outcome of anti-estrogen therapy. *Int. J. Cancer*, **47**, 827-832.

MIZOGUCHI, H., UCHIUMI, T., ONO, M., KOHNO, K. & KUWANO, M. (1990). Enhanced production of tissue-type plasminogen activator by estradiol in a novel type variant of human breast cancer MCF-7 cell line. *Biochim. Biophys. Acta*, **1052**, 475-482.

ORENSTEIN, N.S., BUCZYNSKI, A. & DVORAK, F. (1983). Cryptic and active plasminogen activator by line 10 tumor cells in culture. *Cancer Res.*, **43**, 1783-1789.

OSSOWSKI, L. & REICH, E. (1983). Antibodies to plasminogen activator inhibit tumor metastasis. *Cell*, **35**, 611-619.

PARANJPE, M., ENGEL, L., YOUNG, N. & LIOTTA, L.A. (1980). Activation of human breast carcinoma collagenase through plasminogen activator. *Life Sci.*, **26**, 1223-1231.

PERSKY, B., OSTROWSKI, L.E., PAGAST, P., AHSAN, A. & SCHULTZ, M. (1986). Inhibition of proteolytic enzymes in the *in vitro* amnion model for basement membrane invasion. *Cancer Res.*, **46**, 4129-4134.

REICH, R., THOMPSON, E.W., IWAMOTO, Y., MARTIN, G.R., DEASON, J.R., FULLER, G.C. & MISKIN, R. (1988). Effects of inhibitors of plasminogen activator, serine proteinases, and collagenase IV on the invasion of basement membranes by metastatic cells. *Cancer Res.*, **48**, 3307-3312.

REILLY, D., ANDREASEN, P. & DUFFY, M.J. (1990). Studies on plasminogen activator inhibitor 1 levels in human breast cancer. *Biochem. Soc. Transact.*, **18**, 354-355.

SAPPINO, A.P., BUSSO, N., BELIN, D. & VASSALLI, J.D. (1987). Increase of urokinase-type plasminogen activator gene expression in human lung and breast carcinomas. *Cancer Res.*, **47**, 4043-4046.

SKRIVER, L., LARSSON, J.I., KIELBERG, V., NIELSEN, L.S., ANDREASEN, P.A., KRISTENSEN, P. & DANO, K. (1984). Immunocytochemical localization of urokinase-type plasminogen activator in Lewis lung carcinoma. *J. Cell Biol.*, **99**, 753-758.

SUMIYOSHI, K., BABA, S., SAKAGUCHI, S., URANO, T., TAKADA, Y. & TAKADA, A. (1991). Increase in levels of plasminogen activator and type-1 plasminogen activator inhibitor in human breast cancer: possible roles in tumor progression and metastasis. *Thromb. Res.*, **63**, 59-71.

THORPE, S.M., ROCHEFORT, H., GARCIA, M., FREISS, G., CHRISTENSEN, I.J., KHALAF, S., PAOLUCCI, F., PAU, B., RASMUSSEN, B.B. & ROSE, C. (1989). Association between high concentrations of Mr 52,000 cathepsin D and poor prognosis in human breast cancer. *Cancer Res.*, **49**, 6008-6014.

UCHIUMI, T., MIZOGUCHI, H., HAGINO, Y., KOHNO, K. & KUWANO, M. (1991). Counteraction of estradiol-induced activation of tissue-type plasminogen activator in human breast cancer cell line by an anti-estrogen LY117018. *Int. J. Cancer*, **47**, 80-85.

YAMASHITA, J., HORIUCHI, S., SHIGAKI, N., FUJINO, N. & AKAGI, M. (1984). Estrogen-dependent plasminogen activator in 7,12-dimethylbenz(a)anthracene-induced rat mammary tumors *in vivo* and *in vitro*. *Jpn. J. Cancer Res. (Gann)*, **75**, 681-689.

YAMASHITA, J., HORIUCHI, S., KIMURA, M., NISHIMURA, R. & AKAGI, M. (1986). Plasminogen activator as a functional marker for estrogen dependence in human breast cancer cells. *Jpn. J. Cancer Res. (Gann)*, **77**, 177-181.

YAMASHITA, J., INADA, K., YAMASHITA, S., MATSUO, S., NAKASHIMA, Y. & OGAWA, M. (1992). Specific stimulation by estradiol of tissue-type plasminogen activator production in 7,12-dimethylbenz(a)anthracene-induced rat mammary tumor cells. *Horm. Metabol. Res.*, **24**, 565-569.